# MRI does not detect acetabular osteolysis around metal-on-metal Birmingham THA

**DOI:** 10.1007/s00402-014-2005-9

**Published:** 2014-05-18

**Authors:** Wenzel Waldstein, Tom Schmidt-Braekling, Friedrich Boettner

**Affiliations:** Adult Reconstruction and Joint Replacement Division, Hospital for Special Surgery, Weill Medical College of Cornell University, 535 East 70th Street, New York, NY 10021 USA

**Keywords:** Periacetabular osteolysis, Metal-on-metal, Total hip arthroplasty, Screening, MRI, CT

## Abstract

**Objective:**

Osteolysis has not been recognized as a common failure mode of the Birmingham modular metal-on-metal (MoM) total hip arthroplasty (THA). The clinical value of metal artifact reduction sequence (MARS) magnetic resonance imaging (MRI) to assess the periprosthetic soft tissue is well documented; however, the appropriate image modalities to detect periacetabular osteolysis remain unclear.

**Case summary:**

Eleven patients with periacetabular osteolysis within 3–6 years after uncemented Birmingham modular MoM THA with a synergy stem are presented. All 11 patients received corresponding standardized AP pelvis radiographs, high-quality MARS MRIs and CT scans with a metal artifact reduction sequence. While periacetabular osteolysis around MoM THA was not detected on MARS MRI in ten patients, CT imaging identified osteolysis in all patients. Periacetabular osteolysis appears to be a failure mechanism of the Smith & Nephew Birmingham MoM THA.

**Discussion:**

There is no evidence in the literature to support the effectiveness of MARS MRI to detect periacetabular osteolysis around cobalt chromium alloy metal-on-metal total hip arthroplasties. Osteolysis due to corrosion-related particles seems to be one of the primary modes of failure in modular MoM THA.

**Conclusions:**

MRI is not a sensitive test to identify periacetabular osteolysis. The authors recommend CT for the screening of implants with this failure mode. Our study suggests that patients with a Birmingham modular MoM THA are at increased risk to develop acetabular osteolysis and should be carefully monitored for this failure mode.

## Introduction

The postulated advantages of large metal-on-metal articulations are improved range of motion, increased joint stability [1, 2] and less volumetric wear debris compared to standard metal-on-polyethylene bearing surfaces [3, 4]. Well-designed and properly positioned [5] metal-on-metal hip resurfacings have shown excellent clinical results for selected patients at 10 years [6–8]. However, the outcome of metal-on-metal total hip arthroplasty (THA) has been less predictable and some have been withdrawn from the market because of high failure rates [9]. Modular metal-on-metal (MoM) THAs consist of an acetabular component identical to hip resurfacings, a femoral component as in standard THA and a large (>36 mm) metal head that fits the metal socket. In some systems, the stem and head are connected through a modular sleeve to adjust for different neck lengths.

Early implant failure due to adverse local soft-tissue reactions (aseptic lymphocytic vasculitis-associated lesions (ALVAL) [10]) has been reported for some MoM resurfacings and total hips arthroplasties [11]. Recently, periacetabular osteolysis has been described as a reason for revision in modular MoM THA [12–14]. While the clinical value of metal artifact reduction sequence (MARS) magnetic resonance imaging (MRI) in the assessment of periprosthetic soft tissues is well documented [15, 16]; the best image modality to detect periacetabular osteolysis around metal-on-metal implants remains unclear [13, 17–19].

Considering the excellent track record of the Birmingham hip resurfacing (Smith & Nephew, Memphis, TN) [6, 20] it should be assumed that the Birmingham modular total hip replacement (Smith & Nephew, Memphis, TN), sharing the same design rationale, should also have excellent long-term results.

The purpose of the present case series is (1) to report on 11 patients with periacetabular osteolysis around a Birmingham modular metal-on-metal total hip arthroplasty (Smith & Nephew, Memphis, TN, USA) and (2) to report on the best imaging to detect periacetabular osteolysis around this implant.

## Case report

The current case series presents 11 patients (12 hips) who underwent follow-up with conventional radiographs, CT imaging and MRI imaging at 4–6 year after implantation of an uncemented Birmingham modular MoM THA (Birmingham Hip acetabular cup, Birmingham Hip modular head and Synergy stem; Smith & Nephew, Memphis, TN, USA). These patients are part of a single surgeon follow-up study of which 20 % presented with periacetabular osteolysis. The demographics, implant characteristics and position, and the clinical findings are shown in Table 1. Eight hips were revised at a mean of 53 month after the index procedure. In all revised hips, the modular femoral neck taper junction showed a black color resembling corrosion. The remaining four hips are under follow-up at the time this report was written. All 11 patients received corresponding standardized AP pelvis radiographs, high-quality MARS MRIs at the authors’ institution and CT scans with artifact reduction at an independent radiology practice (Table 2). For the MRI and CT scan interpretations, the official radiographic reports of board-certified radiologists blinded to the reading of the respective CTs or MRIs were used. Metal ion levels (cobalt and chromium) were obtained for all patients. Two representative patients (patients 2 and 8) were selected for further discussion of the clinical workup, and the radiographic and clinical findings.

**Table 1:** Demographics, implant size, implant position, and clinical findings of the 10 patients

Patient	Sex	Age (years)	Side	Head size (mm)	Implant survival (months)	Inclination (°)	Anteversion (°)	Hip pain (VAS)	Cobalt (µg/L)	Chromium (µg/L)
1	Male	84	Left	48	44 (R)	46	23	0	1.5	<1
2	Male	65	Right	48	56 (R)	47	26	5	14.2	Not available
			Left	46	58	46	13	3		
3	Female	71	Left	44	51 (R)	46	23	9	7.4	Not available
4	Male	66	Left	46	74	45	24	0	3.5	2.4
5	Male	45	Right	50	65 (R)	46	14	0	4.8	1.1
6	Male	59	Right	46	53 (R)	45	22	0	1.2	1.1
7	Female	62	Left	42	57	41	21	0	2.4	1.6
8	Female	58	Right	44	49 (R)	50	15	6	6.5	1.1
9	Male	59	Right	48	48 (R)	43	21	2	3.1	1.5
10	Female	53	Left	44	41	44	21	1	4.4	3.9
11	Female	65	Right	46	61 (R)	44	22	0	14.9	12.9

*VAS* visual analog scale,* 0* no pain,* 10* most severe pain, *R* the implant was revised in these patients

**Table 2:** An overview of the official radiographic reports on the assessment of acetabular osteolysis on corresponding CT scans, and MARS MRIs and AP pelvis radiographs is provided for all 101 patients

Patient	Side	CT findings	MRI findings-osteolysis	MRI findings-soft tissue	AP pelvis radiograph findings
1	Left	2 osteolytic lesions anterior acetabulum: 1.3 × 1.8 × 2.0 cm posterior acetabulum: 0.7 × 1.2 cm	No osteolysis	No evidence of abnormalities	No osteolysis
2	Right	2 osteolytic lesions anteromedial acetabulum: 5 × 1 cm	No osteolysis	Moderate-to-severe adverse local tissue reaction	Periprosthetic lucencies in DeLee zone 1 and 2
2	Left	2 osteolytic lesions anterolateral acetabulum: 2.2 × 1 cm	No osteolysis	Mild adverse local tissue reaction	Periprosthetic lucency DeLee zone 1
3	Left	1 osteolytic lesion lateral acetabulum: minimal bony resorption	No osteolysis	Mild-to-moderate bulky proliferative inflammatory response	Periprosthetic lucency in DeLee zone 3
4	Left	1 osteolytic lesion anterior acetabulum: 1.1 × 0.9 × 0.6 cm	No osteolysis	Mild-to-moderate nonspecific synovitis	No osteolysis
5	Right	1 osteolytic lesion superior acetabulum: 3.5 × 2.3 × 6 cm	No osteolysis	Synovial expansion and thickening consistent with adverse local tissue reaction	No osteolysis
6	Right	2 osteolytic lesions anterosuperior acetabulum: 1.2 × 1.2 × 0.8 cm posterior acetabulum: 0.5 × 0.7 × 0.5 cm	No osteolysis	Dehiscent posterior joint capsule	No osteolysis
7	Left	2 osteolytic lesions anterosuperior acetabulum: 1.3 × 1.4 × 1.0 cm anterosuperior acetabulum: 1.1 × 0.9 × 1.2 cm	No osteolysis	No evidence of abnormalities	No osteolysis
8	Right	2 osteolytic lesions anterosuperior acetabulum: 2.1 × 2.1 × 1.6 cm	No osteolysis	Mild intracapsular burden of wear-induced synovitis	Periprosthetic lucency in DeLee zone 1
9	Right	1 osteolytic lesion lateral acetabulum: 1.1 × 1.8 cm	No osteolysis	No evidence of abnormalities	No osteolysis
10	Left	1 osteolytic lesion superior acetabulum: 1.1 × 0.9 × 0.9 cm	No osteolysis	Mild synovitis debris suggestive of an adverse local tissue reaction	No osteolysis
11	Right	1 osteolytic lesion superior acetabulum: 6.1 × 2.6 × 2.7 cm	Focal osseous resorption at the posteromedial aspect of the acetabular component	Mild adverse local tissue reaction	No osteolysis

The MRI was obtained on average 23 days before CT scan. MRI did not detect periacetabular osteolysis as assessed on CT

### Patient 2

**Fig. 1:**
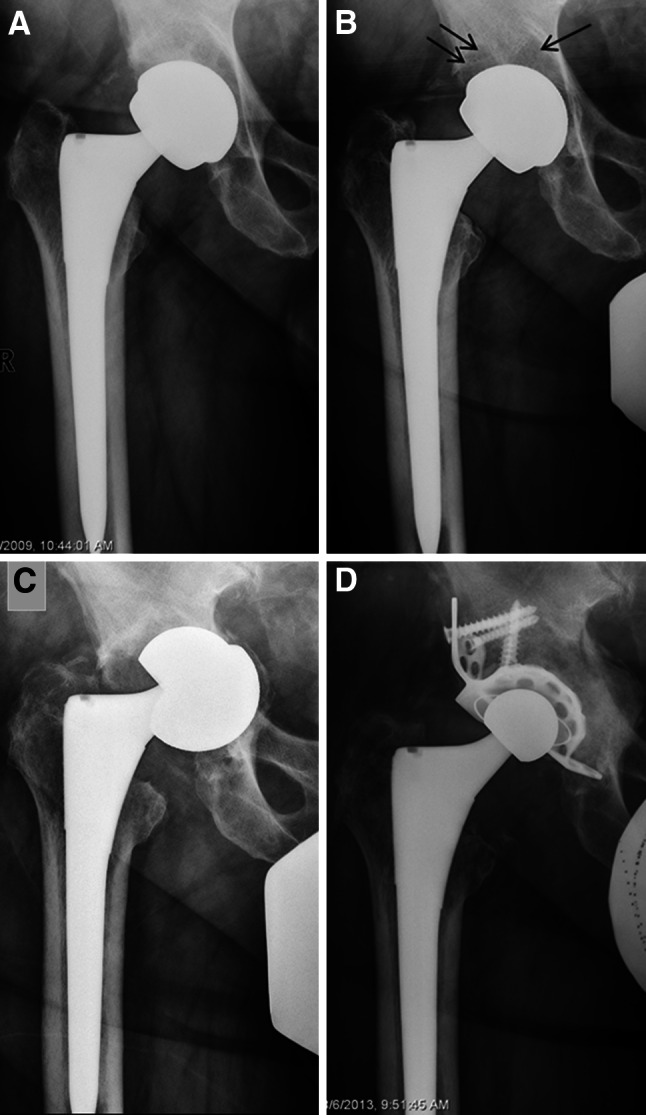
Standardized anteroposterior (AP) pelvis radiographs showing a Birmingham modular metal-on-metal total hip arthroplasty in a 65-year-old man (patient 2): **a** well-aligned components were observed 8 weeks after surgery; **b** periprosthetic lucencies in DeLee zone 1 and 2 were evident 4 months prior to revision surgery (*black arrows*); **c** the loose right cup was seen on the day of revision surgery and **d** AP pelvis radiograph 8 weeks after revision surgery demonstrating high-density bone grafting and an acetabular cage with a cemented liner

Patient 2 was a 65-year-old man who received bilateral MoM THA in 2008. He presented for his follow-up visits at 1 and 5 months postoperative. The components were well aligned and he was pain free (Fig. 1a). The patient presented again for follow-up at 53 months after the index procedure. On the right side, he reported pain in his thigh for the past 3 months. On the left side, he was asymptomatic. The AP pelvis radiograph suggested periprosthetic lucencies in DeLee [21] zone 1 and 2 on the right side and in zone 1 on left side, respectively (Fig. 1b). The CT of the right hip showed an osteolytic lesion extending 5 × 1 cm in the anteromedial acetabulum. On the left hip, CT demonstrated a lesion of 2.2 × 1 cm in the anterolateral acetabulum. The corresponding MARS MRI which was obtained at the same day did not detect any areas of bone resorption in the acetabulum on either side. The patient was scheduled for bilateral hip revision surgery the following months; however, the surgery was postponed because of signs of cardiac ischemia in the preoperative stress test. After successful stent implantation, hip revision surgery was scheduled 3 months later. On the day of revision surgery, an additional AP pelvis radiograph was obtained which showed a loose and displaced right acetabular component (Fig. 1c). Considering the anticipated difficulty of the surgery and the medical condition of the patient, only the right hip was revised at this point. Intraoperatively, there was a significant amount of fluid collection, but no clear evidence of abnormal soft tissue proliferation. The acetabular component was loose and associated with a large acetabular defect medially, anteriorly and superiorly. The acetabular osteolysis required bone grafting and placement of a cage with a cemented liner (Contour Cage, Reflection cemented Cup, Oxinium head; Smith & Nephew, Memphis, TN). The postoperative radiographs, showed well-aligned components (Fig. 1d).

### Patient 8

**Fig. 2:**
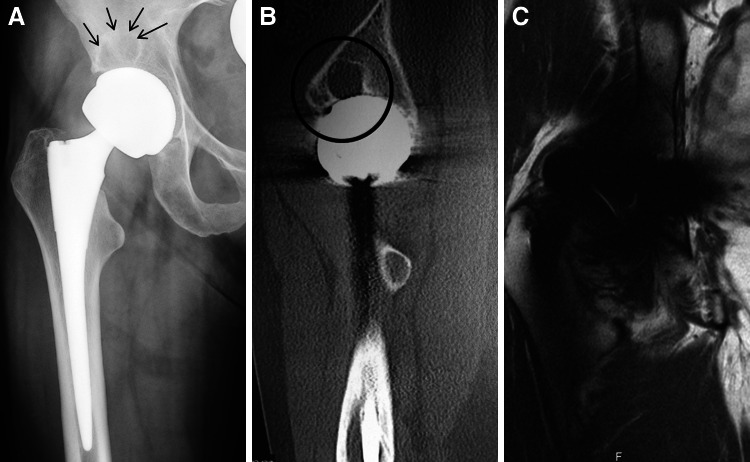
**a** Standardized anteroposterior pelvis radiographs demonstrating a Birmingham modular metal-on-metal total hip arthroplasty in a 58-year-old woman (patient 8) with periprosthetic lucency in DeLee zone 1 (*black arrows*). **b** Corresponding CT with metal artifact reduction showing periprosthetic osteolysis in the acetabular roof. **c** Corresponding MARS MRI with no evidence of acetabular osteolysis

Patient 8 was a 58-year-old woman with right Birmingham modular metal-on-metal THA implanted in 2009. At 30 months, she presented with right hip pain that had progressively worsened over the course of the last year. Her AP pelvis radiograph showed an area of lucency in DeLee zone 1 on the right side (Fig. 2a). The CT scan identified an osteolytic lesion of 2.1 × 2.1 × 1.6 cm in the anterosuperior acetabular roof (Fig. 2b); however, the MARS MRI did not show periacetabular osteolysis (Fig. 2c). Right hip revision surgery confirmed the osteolytic lesion. A thickened synovium was observed but there was no frank evidence of ALVAL. After removal of the well-fixed acetabular component, the defect was bone grafted, and a hemispherical cup was impacted and secured with screws (Trident, Stryker, Mahwah, NJ; Oxinium head, Smith & Nephew, Memphis, TN).

## Discussion

The current study is the first to demonstrate that progressive periacetabular osteolysis should be ruled out in patients with the Birmingham modular metal-on-metal THA (Smith & Nephew, Memphis, TN) and that this failure mode is not detected on MARS MRI. This finding is of high clinical importance as the majority of patients only had mild hip pain and minimal local adverse soft-tissue reactions on MARS MRI and would not have been considered for further aggressive follow-ups. CT imaging identified osteolysis in all patients and allowed for accurate quantification of its size. Periacetabular osteolysis can result in devastating complications as outlined in patient 2 and the authors recommend CT screening for patients with the Birmingham modular metal-on-metal THA. The current findings suggest that CT with metal artifact reduction is a better tool to detect periacetabular osteolysis in MoM THA and should be considered for screening patients with the Birmingham modular metal-on-metal THA and other implants with this failure mode.

Walde et al. [19] compared the accuracy of radiography, CT and MRI in assessing periacetabular osteolysis in standard metal-on-polyethylene THA. The authors utilized a previously described cadaver model [22]. Lesions of varying size were created in the ilium, ischium, pubis and osseous posterior rim and filled with ground beef to mimic granuloma tissue. In their study, MRI detected smaller lesions compared to CT, which was not dependent on the location. Yet CT was more accurate in determining the lesion size. The authors concluded that MRI was the most effective tool to detect bone lesions before they might threaten cup stability. To the best of our knowledge this is the only study in the literature directly comparing the effectiveness of MRI and CT to detect periacetabular osteolysis; however, it focuses on a titanium shell with a plastic liner and its findings might not apply to cobalt chromium alloy metal-on-metal total hip arthroplasties. The authors’ observation has been that for metal-on-metal standard THAs, MRI can fail to detect periacetabular osteolysis. The current findings suggest that CT with metal artifact reduction is a better tool in assessing periprosthetic acetabular osteolysis in MoM THA and to screen implants which are reported to fail secondary to periacetabular osteolysis.

Although, the case series nature of the current paper represents a limitation, osteolytic lesions were detected by MRI in only one patient suggesting that MRI is not a reliable screening tool to assess periacetabular osteolysis in patients with MoM THA. Computed tomography is a better tool to screen patients with implants reported to fail due to periacetabular osteolysis. However, CT screening exposes patients to a 16-fold total effective radiation dose (23 mSv) compared to two views of conventional hip radiographs (1.4 mSv). It must therefore be used with care especially in young female patients. The study also highlights the importance of further investigations comparing the sensitivity and specificity of CT and MRI to detect periacetabular osteolysis in MoM THA.

Previous investigators have described the problems of corrosion at modular femoral neck taper junctions with different metal alloys and the associated increased failure rates [9, 14, 23–30]. Gilbert et al. [25] reported that titanium and its alloys develop a protective layer by passivation from Ti to TiO_2_. Meyer et al. [14] suggested that instability at the taper leads to micromotion which subsequently damages the passivation layer resulting in galvanic corrosion. Micromotion with small amplitudes due to the instability further causes fretting corrosion [14] which results in increased wear particle debris from the taper junction [14, 27, 28]. Meyer et al. [14] reported that 59 of 114 patients with large head modular MoM THA already showed radiographic signs of osteolysis. The analysis of periprosthetic tissue, sampled at revision surgery, revealed that large amounts of titanium or iron were released [14]. The authors concluded that such corrosion leads to a tissue response that induces osteolysis. The tissue reaction is different from ALVAL reactions [10] as observed in other metal-on-metal implants [14].

The observations in the current case series support Meyer’s [14] findings and suggest that the Birmingham modular MoM total hip arthroplasty (Smith & Nephew, Memphis, TN) has a similar failure mechanism. The authors therefore recommend routine CT screening for these patients. The modular neck junctions showed black discoloration in all cases which furthermore supports the concept of osteolysis due to corrosion-related particles as the primary mode of failure in this patient population.

In the majority of cases, the metal ion levels were within normal limits. Only one patient demonstrated an elevated cobalt level (14.2 µg/L) which still seemed relatively low considering that this patient had bilateral MoM implants. Metal ions released in the corrosion process might therefore be different from cobalt and chromium which would explain the low systemic levels of cobalt and chromium in the current study. Our findings are further supported by Meyer et al. [14] who demonstrated that measured levels for cobalt and chromium as well as nickel in the tissues were low in cases with failed modular MoM THA.

The low systemic levels of cobalt and chromium furthermore suggest minimal wear between the metal bearings of the Birmingham standard total hip arthroplasty system which has previously been reported for the Birmingham hip resurfacing system [31].

In conclusion, MRI failed to detect periacetabular osteolysis in patients with the Birmingham modular MoM THA. Although CT exposes patients to ionizing radiation, it has benefits for the screening for osteolysis in patients with this implant. The current case series suggests that the Birmingham metal-on-metal total hip arthroplasty might fail because of corrosion-related osteolysis. This failure mode should also be considered for the modular R3 metal-on-metal cup with a similar metal sleeve (Smith & Nephew, Memphis, TN); however, the authors do not have experience with this implant. Surgeons should screen their patients with this implant carefully for periacetabular osteolysis.
